# Asafoetida exerts neuroprotective effect on oxidative stress induced apoptosis through PI3K/Akt/GSK3β/Nrf2/HO-1 pathway

**DOI:** 10.1186/s13020-022-00630-7

**Published:** 2022-07-06

**Authors:** Qianqian Huang, Chen Zhang, Shi Dong, Junwen Han, Sihao Qu, Tianshu Xie, Haibin Zhao, Yuanyuan Shi

**Affiliations:** 1 Beijing University of Chinese Medicine Affiliated Third Hospital, Beijing, China; 2 School of Life Sciences, Beijing University of Chinese Medicine, Beijing, China; 3 College of Traditional Chinese Medicine, Beijing University of Chinese Medicine, Beijing, China; 4 Beijing University of Chinese Medicine Affiliated Dongfang Hospital, Beijing, China; 5 Shenzhen Research Institute, Beijing University of Chinese Medicine, Shenzhen, China

## Abstract

**Background:**

Alzheimer's Disease (AD) is a serious neurodegenerative disease and there is currently no effective treatment for AD progression. The use of TCM as a potential treatment strategy for AD is an evolving field of investigation. Asafoetida (ASF), an oleo-gum-resin isolated from Ferula assa-foetida root, has been proven to possess antioxidative potential and neuroprotective effects, which is closely associated with the neurological disorders. However, the efficacy and further mechanisms of ASF in AD experimental models are still unclear.

**Methods:**

A cognitive impairment of mouse model induced by scopolamine was established to determine the neuroprotective effects of ASF in *vivo*, as shown by behavioral tests, biochemical assays, Nissl staining, TUNEL staining, Immunohistochemistry, western blot and qPCR. Furthermore, the PC12 cells stimulated by H_2_O_2_ were applied to explore the underlying mechanisms of ASF-mediated efficacy. Then, the UPLCM analysis and integrated network pharmacology approach was utilized to identified the main constitutes of ASF and the potential target of ASF against AD, respectively. And the main identified targets were validated in *vitro* by western blot, qPCR and immunofluorescence staining.

**Results:**

In *vivo*, ASF treatment significantly ameliorated cognitive impairment induced by scopolamine, as evidenced by improving learning and memory abilities, and reducing neuronal injury, cholinergic system impairment, oxidative stress and apoptosis in the hippocampus of mice. In *vitro*, our results validated that ASF can dose-dependently attenuated H_2_O_2_-induced pathological oxidative stress in PC12 cells by inhibiting ROS and MDA production, as well as promoting the activities of SOD, CAT, GSH. We also found that ASF can significantly suppressed the apoptosis rate of PC12 cells increased by H_2_O_2_ exposure, which was confirmed by flow cytometry analysis. Moreover, treatment with ASF obviously attenuated H_2_O_2_-induced increase in caspase-3 and Bax expression levels, as well as decrease in Bcl-2 protein expression. KEGG enrichment analysis indicated that the PI3K/Akt/GSK3β/Nrf2 /HO-1pathway may be involved in the regulation of cognitive impairment by ASF. The results of western blot, qPCR and immunofluorescence staining of vitro assay proved it.

**Conclusions:**

Collectively, our work first uncovered the significant neuroprotective effect of ASF in treating AD in *vivo*. Then, we processed a series of vitro experiments to clarify the biological mechanism action. These data demonstrate that ASF can inhibit oxidative stress induced neuronal apoptosis to foster the prevention of AD both in *vivo* and in *vitro*, and it may exert the function of inhibiting AD through PI3K/Akt/GSK3β/Nrf2/HO-1pathway.

**Supplementary Information:**

The online version contains supplementary material available at 10.1186/s13020-022-00630-7.

## Introduction

Alzheimer's disease (AD) is an age-related and irreversible neurodegenerative disease, characterized by cognitive dysfunction such as progressive learning and memory ability, and there is no effective therapy that can available to reverse the disease progression [1]. It is estimated that by 2050 the number of people with dementia will reach approximately 131.5 million, with the increase of global life expectancy [2]. The progressive accumulation of amyloid beta (Aβ)-containing senile plaques and neurofibrillary tangles formed by tau protein peptide were recognized as two pathological hallmarks of AD [3, 4]. In addition to those two main pathological features, defective anti-oxidant defense systems induced by production of reactive oxygen species (ROS) has been proven in extensive studies to play a critical role in the pathogenesis of AD [5, 6]. Reactive oxygen species (ROS) is a group of highly active molecules that can disrupt cellular redox homeostasis [7], and is considered as the causative factors of degenerative diseases [8]. ROS accumulation will consume the activity and content of some antioxidant enzymes such as Glutathione peroxidase (GSH-PX), Superoxide dismutase (SOD) and Catalase (CAT) [9], induce some lipid peroxidation products such as Malondialdehyde (MDA) and trigger cellular apoptosis. These pathological changes of antioxidant systems and related apoptotic responses are believed to provoke the impairment of cognitive function and the progress of AD.

Nuclear factor erythroid 2-related factor 2 (NRF2), a major transcription factor, is considered to be a key regulator of cellular antioxidant response. Studies have shown that under quiescent conditions, the transcription factor Nrf2 interacts with the actin-anchored protein Keap1, largely localized in the cytoplasm [10]. Upon exposure to oxidative stress or agents that modulate cysteine residues in Keap1, Nrf2 is released from continual degradation by dissociating from Keap1 and translocated into the nucleus, where it stimulates the activation of antioxidant enzymes and its downstream-regulated protein heme oxygenase-1 (HO-1), both known to reduce intracellular ROS [11–13]. Previously, a significant reduction of Nrf2 levels coupled with decreased HO-1 expression was observed in the hippocampus of scopolamine-exposed mice by Wan et al. and the lack of Nrf2 and HO-1 has been shown to accelerate the nerve damage of brain tissues, while the activation of Nrf2 pathway has a neuroprotective effect [14, 15]. In addition, many researches have demonstrated for more than one time that their therapeutic drug enhanced the translocation of Nrf2 into the nucleus, compared with the model group [16]. Thus, all these evidences highlight the protective role of the activation of Nrf2-HO-1 signaling in neurodegenerative conditions, which may offer a potential approach to the alleviation of AD by antioxidants. As more mechanisms of AD were discovered, various therapies have been applied to clinical treatment of AD. However, the treatment relying on single target has not yet achieved a satisfactory therapeutic effect. Due to its multi-component and multi-target characteristics, traditional Chinese medicine has shown obvious potential in the treatment of AD. In addition, compared with other synthetic compounds, Chinese medicine is a natural medicine, which is widely used due to its small side effects, low effectiveness and low toxicity [17].

Ferula assa-foetida belongs to Umbelliferae (Apiaceae) family, an ancient traditional phytomedicine, mainly distributed in the Xinjiang Uygur Autonomous Region of China, India, and Iran [18–20]. Its oleo gum resin, commonly known as Asafoetida (ASF) (named “AWEI” in Chinese Pharmacopoeia) [21, 22], has been report to possess high medicinal value. Traditionally, it has been used in China for thousands of years to treat diseases such as stomach pain and rheumatoid arthritis [23–25]. More and more investigations have revealed some of the effects of ASF on nervous system function, especially in terms of neuroprotective and nerve stimulating effects. Moghadam et.al investigate the effects of asafoetida extracts on pyridoxine-induced neuropathy in mice and display neuroprotective effects through regulating axonal regeneration and remyelination of lymphocyte infiltration [26]. In addition, Yan et.al found in ischemic brain injury diseases, the active ingredients of asafoetida can inhibit the production of ROS and increase the activity of NADPH-oxidase (NOX) to exert neuroprotective functions[27]. These findings explained the beneficial effects of asafoetida as s a potential therapy for neurological diseases. Also, Modern pharmacological evidences have shown the sesquiterpene coumarins, steroidal esters, volatile oils, are the characteristic components of the resin of Ferula assa-foetida [28, 29], which have various pharmacological activities such as anti-inflammation [30], anti-neurodegeneration, anti-oxidation and anti-apoptotic [31]. Taken together, reports from traditional usages and these new findings show that Asafoetida may exert neuroprotective effects in *vitro* or in *vivo*. Those evidence further support its potential to treat AD, but the efficacy and further mechanisms of ASF in AD experimental models are still unclear. Here, we hypothesize that ASF can treat AD by regulating oxidative stress and neuronal apoptosis to slow down cognitive impairment.

## Materials and methods

### Drugs and reagents

Scopolamine hydrobromide trihydrate was purchased from Aladdin Reagent Co., Ltd. (Batch No. 6533-68-2; Shanghai, China). Donepezil Hydrochloride was purchased from Eisai Pharmaceutical Co., Ltd. (Batch No.2008001; Jiangsu, China). The regent test kits used in the study including Acetylcholine (Ach) assay kit (CAT: A105-1-2), Acetylcholinesterase (AchE) assay kit (CAT: A024-1-1), Glutathione peroxidase (GSH-PX) assay kit (Colorimetric method) (CAT: A005-1-2), Superoxide dismutase (SOD) assay kit (CAT: A001-3-2), Malondialdehyde (MDA) assay kit (TBA method) (CAT: A003-1-2), Cell Malondialdehyde (MDA) assay kit (Colorimetric method) (CAT: A003-4-1), Reduced glutathione (GSH) assay kit (CAT: A006-2-1) and were purchased from Nanjing Jian Cheng Bioengineering Institute. (Jiangsu, Beijing). Reactive oxygen species (ROS) assay Kit (CAT: S0033S), Catalase (CAT) assay kit (CAT: S0051) was purchased from Shanghai Biyuntian Biotechnology Co., Ltd. (Shanghai, China). Annexin V-FITC/PI staining assay (CAT: 640,914) was purchased from BioLegend, Inc. (USA). The RPMI-1640 medium and trypsin were purchased from Corning Incorporated, NY, USA. Horse serum, fetal bovine serum and penicillin-streptomycin were purchased from Gibco, Grand Island, NY, USA. TUNEL Kit (Cat. No.: G1501), Citric Acid (PH6.0) Antigen Retrieval Solution (Cat. No. G1202), EDTA Antigen Retrieval Solution (pH 8.0) (Cat. No. G1206), Hematoxylin Blue-Returning Solution (Cat. No. G1340), Ultrasound Clean quick-drying glue (Cat. No. G1404) and DAB color reagent (Cat. No. G1211) for immunohistochemistry kits were purchased from Servicebio. The antibody used in this study are as followed: PI3K (67,071-1-Ig, proteintech,1:5000), anti-AKT1(phospho S473) (ab81283, abcam, 1:3000), AKT1(phospho S473) (ab179643, abcam, 1:5000), NRF2 (16,396-1-AP, proteintech, 1:), phosphor-GSK3β Phospho-GSK3β (Ser9) (67,558-1-Ig, proteintech, 1:3000), GSK3β (22,104-1-AP, proteintech, 1:3000), BAX (50,599-2-Ig, proteintech, 1:3000), BCL2 (26,593-1-AP, proteintech, 1:3000), GAPDH (ab8245, abcam,1:40,000).

### Preparation of ASF extracts

Asafoetida is an oleo-gum-resin isolated from Ferula assa-foetida root [20]. Raw medicinal materials were purchased from Beijing Dongzhou Traditional Chinese Medicine Clinic Co., Ltd. (Batch No. 190414002; Beijing, China) and identified by Prof. Yuanyuan Shi. The extraction process of ASF refers to previous publications. Briefly, the collected ASF gum were first dried into powders in a mortar and then 300 ml (10 times volumes) of 95% ethanol was added. After refluxing and extracting at 85℃ for 2 h, the supernatant was filtered, and the remaining substances were extracted again according to the above steps. The two filtrates were combined, and then concentrated to 30 ml by rotavapor apparatus. The freshly prepared asafoetida extracts was diluted with distilled water into different doses, and sealed at 4 °C for further experiments. For vitro delivery, 10 mg of asafoetida gum powder was dissolved in DMSO (1 ml) and was ultrasonically extracted for 1 h, then centrifuged at 10,000 rpm for 10 min. Eventually, we collected the supernatant and then diluted the supernatant with DMEM medium to various concentrations for further cell processing.

### Animal

8-week-old C57BL/6 N male mice(20 ± 2 g) were purchased from Charles River Laboratories (animal license: SCXK (JING) 2016–0006). All mice were housed at room temperature of 24 ± 2 °C and the light/dark cycle of 12 h:12 h. The animals were assigned randomly to 5 groups (15 mice per group): control group, model group (scopolamine hydrobromide of 3 mg/kg/day), ASF-L group (Asafoetida extracts of 150 mg/kg/day), ASF-M group (Asafoetida extracts of 75 mg/kg/day), ASF-H group (Asafoetida extracts of 37.5 mg/kg/day). Administration dose of ASF was decided with reference to human daily dose recommended form Chinese Pharmacopoeia 2020 and calculated from the formula that the mouse equivalent dose (g/kg) = human dose (g/kg) × (human Km = 37)/(mouse Km = 3). All mice were given intragastric administration for 14 consecutive days. On the 15th day of administration, one hour after ASF treatment, behavioral tests were conducted. Mice were administrated intraperitoneally with scopolamine (3 mg/kg) 15–20 min before behavioral test. After the final administration, all mice were euthanized. Then, the brains were removed, and then the hippocampus tissues were isolated, quickly collected on ice, then stored at – 80 °C for further experiment. For immunohistochemistry and TUNEL assay, brains were harvested after perfusion with 4% paraformaldehyde (PFA).

### Morris water maze (MWM)

The Morris water maze include the first 5 days of positioning navigation experiment and the last day of space exploration experiment. A 90 cm diameter water tank was divided into four equal quadrants. A transparent plastic platform with a diameter of 10 cm was put under water and TiO2 (Beijing Tyco Beauty Biotechnology Co., LTD., Beijing, China) was integrated into the water to hide the platform. During the first 5 days of training, a quadrant was arbitrarily selected, and each mouse was placed facing the pool wall into the water to practice swimming for 60 s. The time for the mouse to search the target platform within 60 s was defined as escape latency. If the mouse fails to climb on the platform within 60 s, the experiment ends, and the mouse was guided to stand on the platform for 10 s. On the last day, the platform was removed and the swimming time of the mice within 60 s was recorded.

### The step-down passive avoidance (SDA)

The step-down passive avoidance (SDA) test was performed to detect the memory retention ability of mice. A plexiglass chambers was built by several parallel steel bars with electric shock devices at the bottom, and an insulated wooden platform was placed in the corner. On the first day, the mice were allowed to take an adaptive exploration for 3 min in the chambers during the active electric shock device. After 24 h, the escape latency of the mouse (Recorded as the time of mice to jump down the platform for the first time) and the number of errors (Recorded as the number of mice touching the ground) were recorded within 300 s.

### Novel object recognition (NOR)

Novel Object Recognition (NOR) test was performed to detect the short-term memory ability of mice. The entire experiment was performed in a dark environment. Two identical objects, Object A and Object B, were placed in a 40*40*40 cm box and mice were then placed in the box and allowed to explore freely for 5 min before taken out. 24 h later, the object B was replaced by object C. with different appearance. Mice touching or sniffing within 2 cm were considered exploration [32]. The time for the mouse to explore Object A and Object C is recorded as TA and TC. The recognition index is calculated as the ratio TC/(TC + TA). After each rat completes the test, clean the box with 75% ethanol to eliminate the smell of the last rat [33].

### Determination of acetylcholinesterase (AchE) activity and acetylcholine (Ach) level from the mice brain

After the behavioral test, the hippocampus tissues dissected from brain were homogenized on ice and centrifuged at 12,000×*g* for 10 min at 4 °C. Hippocampal Ach content and AchE activity were detected in accordance with the instructions of the Ach assay kit and AchE activity assay kit.

### Nissl staining

Paraffin sections were deparaffinized in xylene for 10 min for three times, then soaked 5 min in 100% ethanol, 2 min in 90% ethanol, 2 min in 70% ethanol and finally soaked in distilled water. The processed sample was stained with Nissl staining solution for 5 min, then washed twice by distilled water, once by 95% ethanol and twice by 70% ethanol. Then the Nissl neurons were observed under light microscopy.

### Immunohistochemistry

For immunohistochemistry assay, the hippocampal tissue slices were frozen and sectioned with a cryostat microtome (8 µm). After that, endogenous peroxidase activity was blocked with 3% H2O2 solution after antigen retrieval. Then, the slides were block with 10% normal goat serum for 1 h. Primary antibodies were applied overnight at 4 °C followed by the incubation of HRP-conjugated secondary antibody for 1 h at room temperature. The slides were visualized by DAB and counterstained with hematoxylin. Images were captured by an Olympus BX51 system and analyzed by Image J software (National Institutes of Health, USA).

### TUNEL assay

To visualize DNA fragmentation in the brain tissues of the mice, we performed TUNEL staining using an In Situ Cell Death Detection Kit (Roche Diagnostics, Risch-Rotkreuz, Switzerland) according to the manufacturer’s protocol. Briefly, brain tissues were fixed in 4% formaldehyde and embedded in paraffin wax. Five micrometer sections of the embedded material were then cut and washed for 30 min in PBS. The sections were then incubated with proteinase K (100 mg/mL), and then rinsed again. Next, they were incubated in 3% H2O2, permeabilized with 0.5% Triton X-100, rinsed again, and incubated in the TUNEL reaction mixture. The sections were rinsed and visualized using Converter-POD with 0.03% DAB, counterstained with Cresyl violet, and mounted onto gelatin-coated slides. The slides were air-dried overnight at room temperature and cover-slipped using Permount mounting medium. The density of apoptotic cells was detected by immuno-fluorescent microscopy (TCS SP5, Leica, Mannheim, Germany) by counting the green-stained cells.

### Cell culture and treatment

PC12 cell line was purchased from Cell Bank of Chinese Academy of Sciences, (Beijing, China) and cultured with RPMI-1640 medium (Geibo, CAT: 11,995–065) containing 10% (v/v) heat inactivated horse serum fetal, 5% (v/v) fetal bovine serum (FBS), 1% penicillin (100 U/ml), and 1% streptomycin (100 ug/ml). Cells of passages 10-passages 20 were used for experiments. To identify the neuroprotective effects of ASF on H_2_O_2_-induced PC12 cells, we first pretreated PC12 cells with different concentrations of ASF (0.1, 1, 10 mg/ml) for 24 h and then subjected to the optimal dose of H_2_O_2_ (175 μmol/ml) for another 4 h.

### Cell viability assay

The Cell counting Kit-8 assay (Beyotime, Haimen, China) was used to detect the cell viability for of PC12 cells with ASF and H_2_O_2_ treatment. In brief, PC12 cells in the logarithmic growth phase were seeded into 96-well plate at a density of 1 × 10^4^ cells/well overnight. Then, ASF of different concentrations (0.1 μg/ml, 1 μg/ml, 10 μg/ml, 50 μg/ml and 100 μg/ml) were added for further culture for 24 h, then different concentrations of H_2_O_2_ were added for 1–4 h. After intervention, the medium was removed and replaced with fresh medium with 10ul CCK reagent for each well. The plate was maintaining at 37ºC in a humidified 5% CO2 atmosphere for 1 h, then the OD number was read under the 450 nm wavelength, respectively.

### Determination of catalase (CAT), malondialdehyde (MDA) and superoxide dismutase (SOD), reduced glutathione (GSH), GLUTATHIONE peroxidase (GSH-PX)

The hippocampus of mice was collected and homogenized with tenfold volume of RIPA buffer. After centrifuge, the upper liquid was obtained and used to detect the activities of SOD, GSH-PX and the level of MDA in the hippocampus according to the manufacturer's protocols of the commercial kits.

The PC12 cells were seeded and cultured in 6-well plates overnight, then pretreated with different dose of ASF (0.1, 1, 10 mg/mL) for 24 h and accompanied by stimulation with H_2_O_2_ (175 μm) for 4 h. After intervention, the supernatant of cell homogenates was collected with 200μL RIPA lysis buffer in each well. Then, the levels of MDA, GSH and the activities of SOD, CAT in the cells were determined according to the corresponding manufacturer's instructions of the assay kit.

### Reactive oxygen species (ROS) assay

PC12 cells were harvested after 24 h of ASF pretreatment and the following 4 h of H_2_O_2_ stimulation. Firstly, DCFH-DA was diluted with serum-free medium at a ratio of 1:1000. Subsequently, the cells were washed with PBS for two times and suspended in DCFH-DA, then incubated at 37 °C for 20 min, and stimulated with H_2_O_2_ for another 4 h. After incubation, PC12 cells were washed three times with medium to remove the extra DCFH-DA that have not entered the cell. Then it was photographed by fluorescence microscope. To quantify the level of ROS, the cells was finally resuspended in 300 μL PBS and analyzed by flow cytometer with excitation and emission wavelengths of 484 and 530 nm, respectively.

### Apoptosis assay by flow cytometer

We used Annexin V-FITC/PI staining assay to detect cell apoptosis rate. The PC12 cells were pre-treated with ASF (10, 1,0.1 μg/mL) for 24 h and H_2_O_2_ for 4 h. After incubation, the cells were harvested and washed twice with PBS, then the cells were resuspended with 100 μL binding buffer followed by adding Annexin V-fluorescein isothiocyanate (5 μL) and PI (10 μL). After mixing and reacting in the dark of room temperature (RT) for 15 min, the cell was centrifuged and resuspended in 400 μL PBS for flow cytometry assessment. The A008 flow cytometer (Beckman Coulter, China) was applied to detect and analyze the stained PC12 cells. The apoptosis rate was recorded as the percentage of apoptotic cells to the overall number of cells.

### Immunofluorescence staining

PC12 cells were seeded in confocal dishes (0.4 million cells per dish) overnight to reach 70–80% confluency on the subsequent day. PC12 cells were treated as described previously. After incubation, the cells were fixed with 4% paraformaldehyde (PFA) for 10 min at RT. After fixation, cells were washed with PBS for three times (10 min each time) and then permeabilized in same volumes of 0.1% Triton X-100 for 10 min at RT. Cells were then washed as previously described and blocked in 1% BSA for 1 h at RT. After blocking, Nrf2 antibody (dilution of 1:200 in 1%BSA in PBS) was added and incubated overnight at 4 ℃. On the next day, cells were washed 5 min × 3 times in PBS then incubated with secondary antibodies diluted of 1:200 in 1%BSA in PBS for 50 min. Then, DAPI (1:1000 dilute) was added for 10 min at RT. The cells then were washed with PBS 5 min × 3 times. The immunofluorescence staining was observed and images were obtained using the inverted fluorescence microscope (CKX4, OLYMPUS, Japan).

### Western blotting

Treatment of PC12 cells as previously described. The protein of PC12 cells were harvested by RIPA buffer (APPPLYGEN, CAT: C1055) covering phosphatase and protease inhibitor. The protein of hippocampus tissue from *vivo* study was harvested by smashing frozen tissue and adding RIPA buffer. The protein concentration of tissue homogenates and cell lysates were measured with a BCA protein assay kit (Applygen Technology Co., Ltd. Beijing, China). The protein extracts from cells and hippocampus tissue with equal protein concentration was separated by SDS-PAGE on 8–12% gel and transferred to PVDF membrane. And then 5% non-fat powered milk were used to blocked the membrane for 3 h at room temperature. Afterwards, the membranes were then incubated with the primary antibody and the corresponding secondary antibody. The protein bands were quantified by Image-Pro Plus 6.0 software.

### UPLC and MS Conditions

Dionex Ultimate 3000 UPLC system was used to analyze the chemical components of ASF. BDS HYPERSIL C18 column (150 mm × 2.1 mm, 2.4 μm; Thermo Fisher Scientific) eluted with a mobile phase of acetonitrile (A) and 0.1% formic acid (B) was applied for Chromatographic separations. The flow rate of the wash liquid was 10.0 μL/s, and the duration was 20 s. The eluent was washed at a flow rate of 0.3 mL/min, and the column temperature was 45 ℃. The gradient started run at 95% eluent B for 3 min, then decrease to 5% eluent B for 45 min, finally, increase to 95% eluent B for 5 min.

Thermo Q-Enactive Orbitrap Mass Spectrometer (Thermo Fisher) was employed for Mass Spectrometer (MS) detection. Under the controlled of Xcalibur 2.1, LTQ-Orbitrap XL (Thermo Fisher Scientific, San Jose, CA, USA) was operate by electrospray ionization source (ESI) in positive and negative ion modes. In the positive ion models, the sheath gas was N2, tube lens was 110 V, the source temperature was 350 °C, and the source voltages was 4 kV. In the negative ion model, the sheath gas was N2, tube lens was 110 V, the source temperature was 350 °C, and the source voltages was 3 kV. The full-scan mass spectrum was recorded in m/z 120–1800.

### The prediction of ASF related targets

Firstly, the raw files obtained by Liquid Chromatography Mass Spectrometry (LC/MS) were processed using Xcalibur version 2.7 software. The constituents of Asafoetida extracts found in UHPLC were compared with TCMSP database (https://old.tcmspe.com/tcmsp.php). Then, Swiss Target Prediction database (http://www.swisstargetprediction.ch/) was used to screen the predicted proteins targeted by Asafoetida extracts. To pick more realizable prediction results, the species was set to homo sapiens, and the screening condition was set to probability > 0.

### Prediction of the putative targets of Alzheimer disease

Using "Alzheimer disease" as keyword, we collect known AD treatment targets from GeneCards (http://www.genecards.org/), OMIM database (http://www.omim.org/), DrugBank database (https://www.drugbank.ca/), DisGeNET (http://www.disgenet.org, version 5.0).

### Construction of a Protein–Protein Interaction (PPI) network of intersection targets between compounds and AD targets

After excluding duplicate data, the shared overlap of verified constituents target of ASF and the therapeutic targets of AD were mapped using Venny 2.1 (bioinfogp.cnb.csic.es/tools/venny/). Then, All the intersection targets were put into Retrieval of Interacting Genes (STRING) string database (www.string-db.org/) with the required score of medium confidence (0.400). Then, the output result was imported to Cytoscape 3.8.2 software to visualize the PPI network. And, the network topology parameters (“betweenness centrality”, “closeness centrality”, and “degree”) were calculated to screen out which targets may have critical roles.

### Analyses of pathway enrichment and construction of the constituents–targets–pathway network

To elucidate the biological functions of the potential targets that involved in ASF extracts-mediated AD treatment, Gene Ontology (GO) annotation analysis containing the biological process (BP), molecular function (MF), cellular components (CC) and Kyoto Encyclopedia of Genes and Genomes (KEGG) enrichment analysis were performed using the Database for Annotation, Visualization and Integrated Discovery (David, www.david.ncifcrf.gov/). All term of GO and KEGG in these databases were considered significant with *P* < 0.05. The network-visualization software Cytoscapev3.8.2. was utilized to construct the constitutes-targets-pathways network of ASF in treating AD. Then the node degree was calculated, and the node whose degree value is greater than twice the median value of all network nodes was regarded as the hub.

### RT-qPCR analysis

The RNA of PC12 cell and hippocampus tissue was extracted by SteadyPure Universal RNA Extractin Kit (Accurate Biotechnology, China). A200 Gradient themal cycler (Long Gene, China) was utilized to reverse transcription of RNA into the cDNA. Real-time amplification of complementary cDNA was measured by QuantStudio6 Flex Real-time fluorescence quantitative PCR instrument on CFX-96 system with SYBR Green Master mix. GAPDH was used as an endogenous control to balance the differences between samples. And final 2 ^ -delta CT method was used to quantify the samples. The primers of animal and cell used are shown in Additional file 1: Table S1.

### Statistical analysis

Student's unpaired t-test was applied to the comparison between two groups. One-way analysis of variance (ANOVA) with post hoc tests was used to examine the significant differences among multiple groups. Two-way ANOVA analysis was used for statistics on the results of the water maze experiment. All data are presented as Mean ± Standard Error, and p ≤ 0.05 was considered statistical significance.

## Result

### ASF improved scopolamine-induced learning and memory impairments in mouse brain

**Fig. 1:**
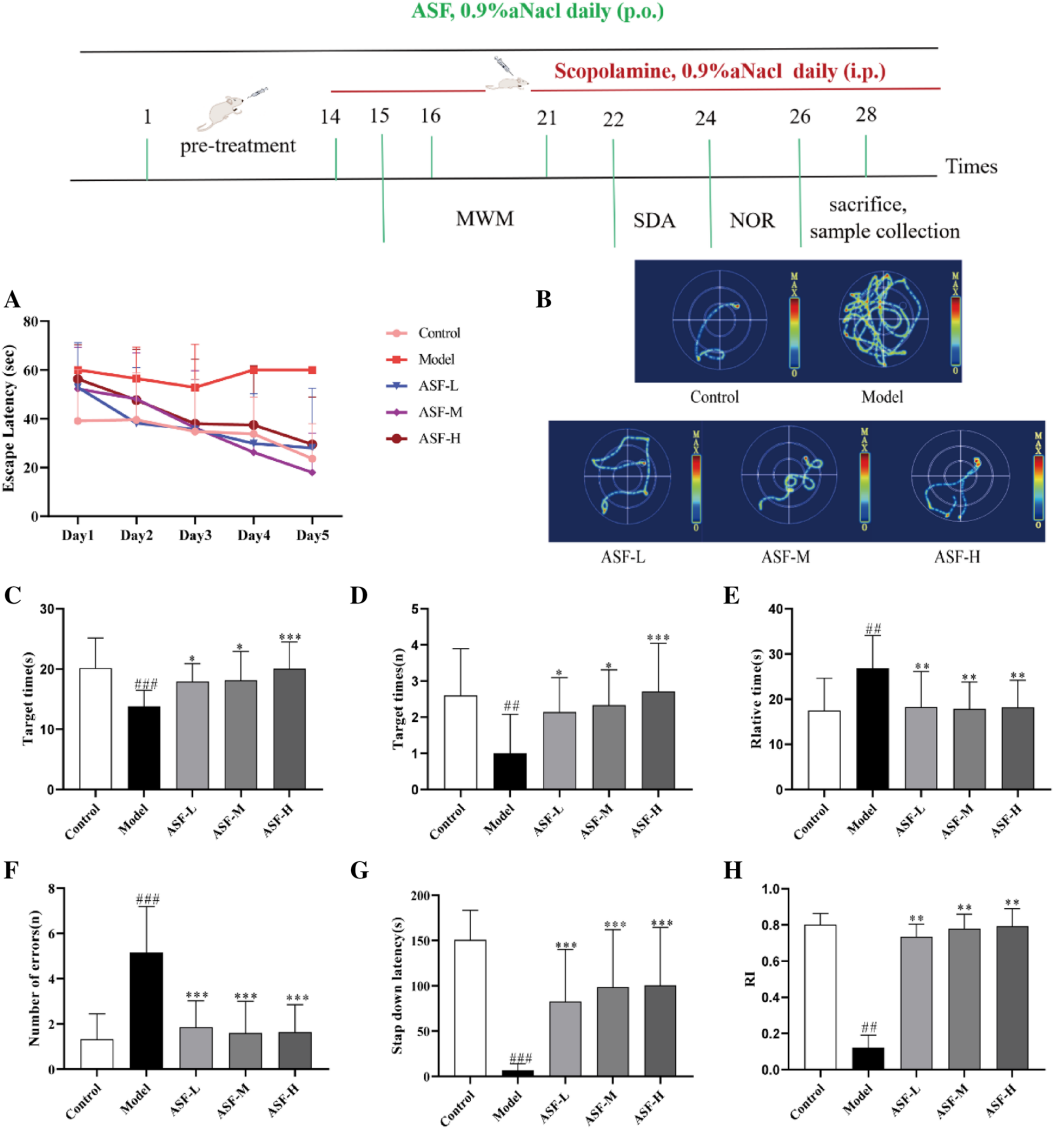
Neuroprotective effect of ASF extracts on learning and memory deficits in scopolamine-treated mice. The escape latency during positioning navigation test (the first 5 consecutive days training) (**A**), Representative swimming tracks from all groups in the water maze test (**B**), The escape latency to find hidden platform (**C**), the numbers of crossing (**D**), retention time spent in relative target quadrant (**E**) on the last day during Morris water maze; The number of errors and latency in step-down passive avoidance test (**F**, **G**); The exploration time percentage of novel objects (Recognition Index RI) in novel object recognition abilities (**H**). Data were expressed as mean ± SE (n = 15). ^##^*p* < 0.01 and ^###^*p* < 0.001 compared to the control group; *p < 0.05, **p < 0.01 and ***p < 0.001 compared to the scopolamine-treated group

To assess the neuroprotective effect of ASF extracts in *vivo*, a mouse model of scopolamine-induced cognitive impairment was constructed. Firstly, the Behavioral tests including Morris Water Maze (MWZ), Step-Down Passive Avoidance (SDA), Novel Object Recognition (NOR) were performed to detect the learning and memory abilities of mice. In Morris Water Maze test, compared with the control group, the escape latency of model group was significantly increased on the first 5 days, while ASF extracts administration in dose of 37.5 mg/kg, 75 mg/kg and 150 mg/kg ameliorated the scopolamine-induced learning deficit (Fig. 1A). In the space exploration test on the last day, Mice in ASF group spent more time in the target quadrant area (Fig. 1B), more crossing numbers in platform area (Fig. 1C), and less time in the relative platform quadrant (Fig. 1D) compared with the model group. In the SDA test, we detected that the treatment of ASF extracts notably recovered scopolamine-induced shorter step-down latency in a dose-dependent manner (Fig. 1E) and a greater number of errors in mice compared with model group (Fig. 1F). In addition, we also found increased RI following scopolamine exposure after ASF extracts. These data suggested that ASF extracts effectively attenuated the memory loss and learning impairment in scopolamine induced AD mice model.

### ASF ameliorated scopolamine-induced neuronal injury in mouse brain

**Fig. 2:**
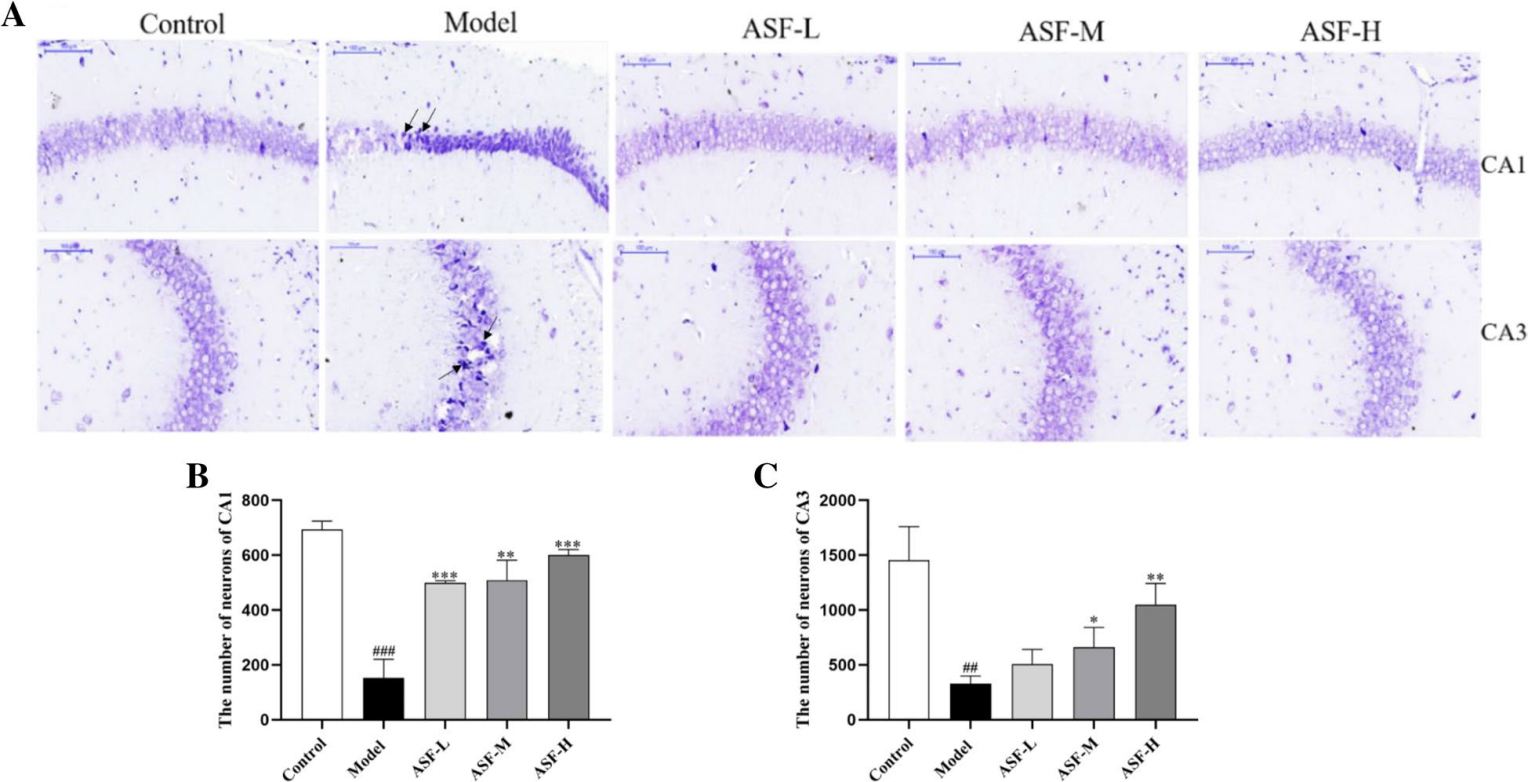
Neuroprotective effect of ASF extracts on hippocampal neuron damage in scopolamine-treated mice. Representative Nissl staining images of the CA1 and CA3 regions of the hippocampal (100x) (**A**); Quantitative analysis of the normal neuron of CA1 and CA3 (**B**, **C**). Data were analysed as mean ± SE (n = 3). ^#^*p* < 0.05 and ^###^*p* < 0.001 compared to the control group; **p < 0.01 compared to the scopolamine-treated group

Histopathological changes in the hippocampus tissue, which is classically associated with disease progression, were assessed by Nissl staining. As report in Fig. 2A, in the control group, the pyramidal neurons in the hippocampal CA1 region and CA3 region were clearly discernible and neatly arranged, with abundant Nissl bodies in the cytoplasm. In the hippocampal of scopolamine-treated mice, the neurons were significantly shrunken, irregularly arranged, and their nuclei were pyknotic and hyperchromatic, which indicated that neurons were diffusely deteriorated or dead and great quantity Nissl bodies were lost in these neurons.

Compared with the control group, the number of pyramidal neurons in the model group significantly decreased, while the pyramidal neurons were better arranged, and the number of the pyramidal neurons was significantly increased in the different doses of ASF extracts group (Fig. 2B, C), suggesting that ASF extracts can alleviate scopolamine-induced pathological damage of mouse hippocampus.

### ASF ameliorated scopolamine-induced cholinergic system dysfunction in mouse brain

**Fig. 3:**
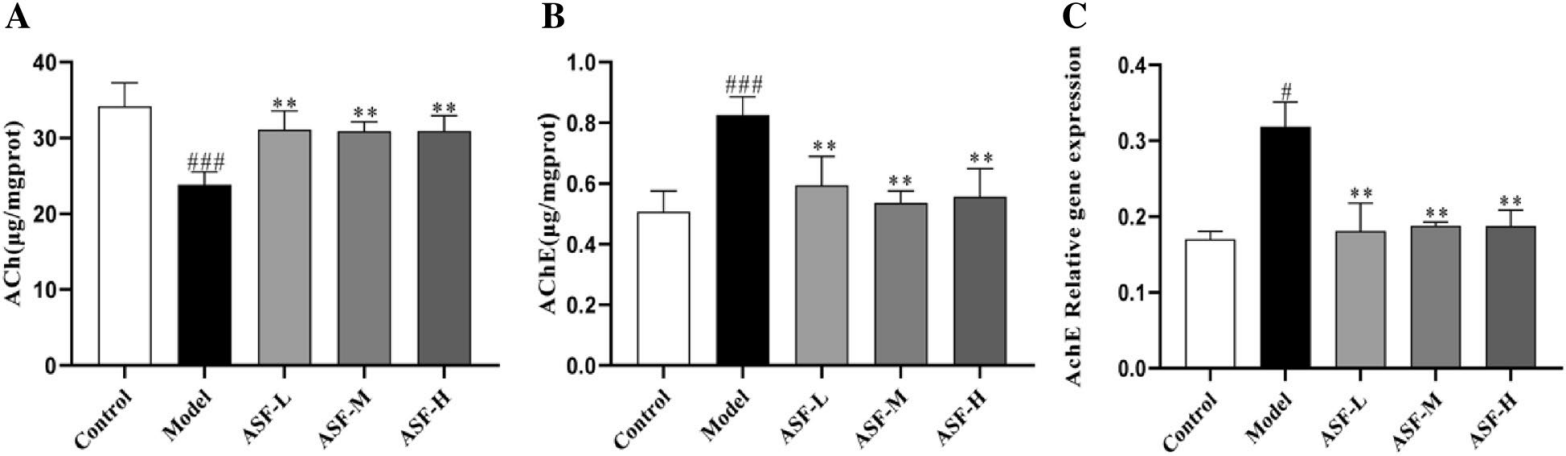
Ach content (**A**) and AchE activity (**B**) were measured according to assay kit instructions. The mRNA expression of AchE in hippocampus tissue were detected by RT-qPCR (**C**). Data were analysed as mean ± SE (n = 3). ^#^*p* < 0.05 and ^###^*p* < 0.001 compared to the control group; **p < 0.01 compared to the scopolamine-treated group

Acetylcholine (Ach), the first identified neurotransmitter, plays an important role in hippocampal memory function and deeply involved in the pathogenesis of AD. Acetylcholinesterase (AchE) is an enzyme that metabolizes the Ach at synaptic cleft, resulting in cognitive impairment. To further assess the neuroprotective of ASF extracts on scopolamine induced hippocampal neuron damage, the acetylcholine (Ach) content and acetylcholinesterase (AchE) activity were evaluated to assess the degree of cholinergic dysfunction. Scopolamine, a muscarinic cholinergic receptor antagonist, can cause impairment of cholinergic transmission resulting in cognitive deficits [34, 35]. As expect, the reduced content of Ach (Fig. 3A), increased AchE activity (Fig. 3B) and elevated AchE gene expression level (Fig. 3C) were observed in hippocampus tissue after scopolamine exposure compared with control group, which were reverted by ASF extracts administration in a dose-dependent manner.

### ASF ameliorated scopolamine-induced impairment of postsynaptic transmission in mouse brain

As a postsynaptic muscarinic receptor blocker, scopolamine can also lead to impaired postsynaptic transmission leading to cognitive deficits. To explore whether ASF could also improve learning and memory by improving postsynaptic transmission, we performed immunohistochemistry to examine the expression of PSD95 and SYN in mouse tissue. The two synapse-related proteins play a role as a "bridge" in the synaptic connection, and the detection of the protein levels of SYP and PSD95 can estimate synaptogenesis and reflect the efficiency of synaptic transmission, which is an important indicator of the level of synaptic remodeling [36, 37].

**Fig. 4:**
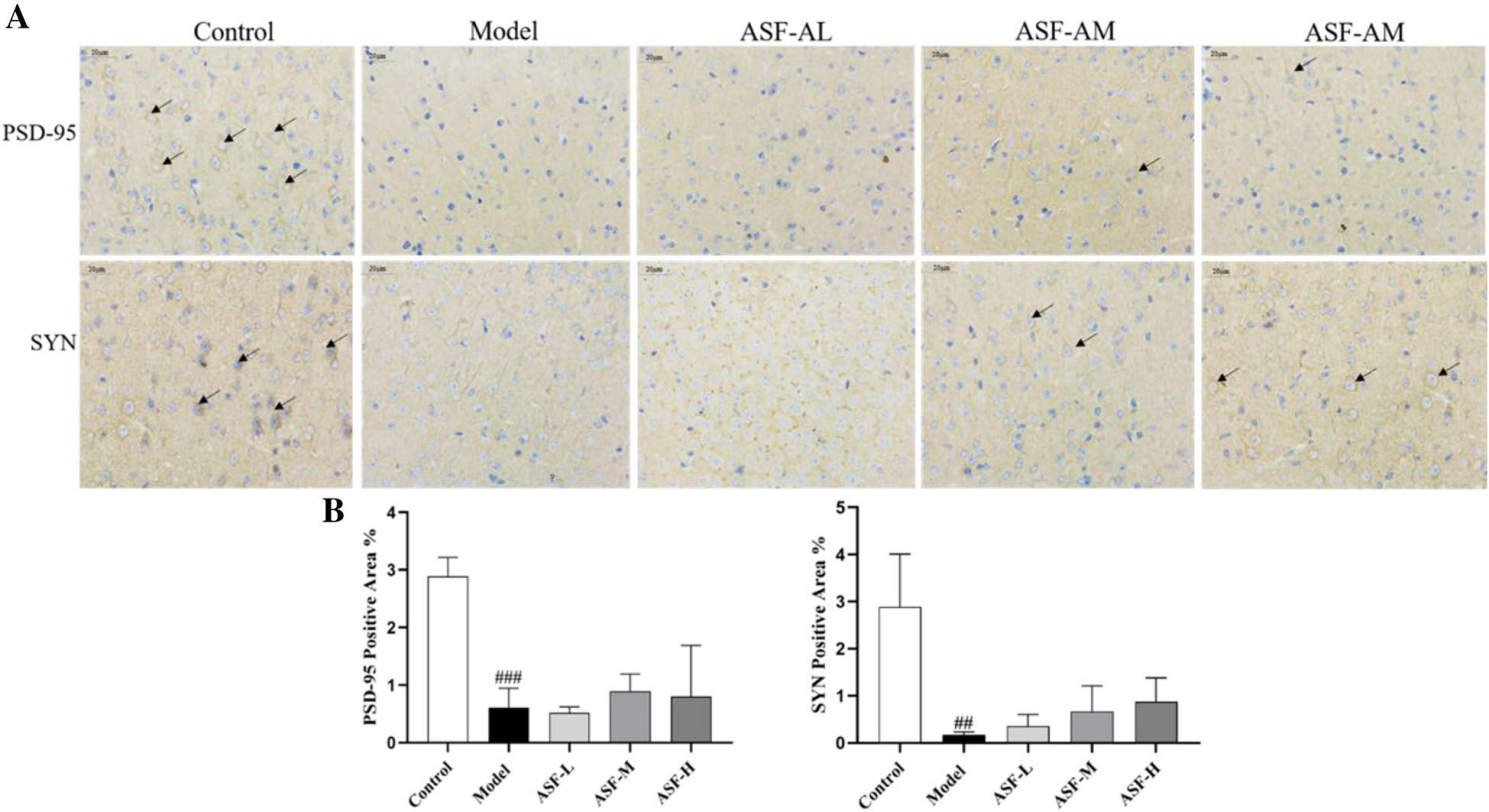
Immunohistochemistry staining assay displayed PSD-95 and SYN positive cells of mouse brain tissue after scopolamine stimulation. The black arrow pointed to positive cells. The scale bar is labeled as 20 μm. The Representative images of immunohistochemistry staining in each group **(A)**. The quantitative percentage of positive cells in total cells of PSD-95 and SYN was assessed in each group **(B)**. Data are shown as mean ± SD, n = 3. #*p* < 0.05, ##*p* < 0.01 and ###*p* < 0.001 compared to the control group; *p < 0.05, **p < 0.01 and ***p < 0.001 compared to the scopolamine-treated group

Immunohistochemical results demonstrated that the positive expression of PSD-95 and SYN was mainly located in cell membranes (yellow brown granules). Compared with the control group, the intercellular space of neurons in the model group increased and the positive expressions of PSD-95 and SYN were significantly decreased in the brain of scopolamine-treated mice, while the positive expressions of PSD-95 and SYN were not significantly increased in the ASF low, medium and high dose groups (Fig. 4A, B).

### ASF protects against scopolamine-induced oxidative stress and the expression of apoptosis-related proteins in mouse brain

**Fig. 5:**
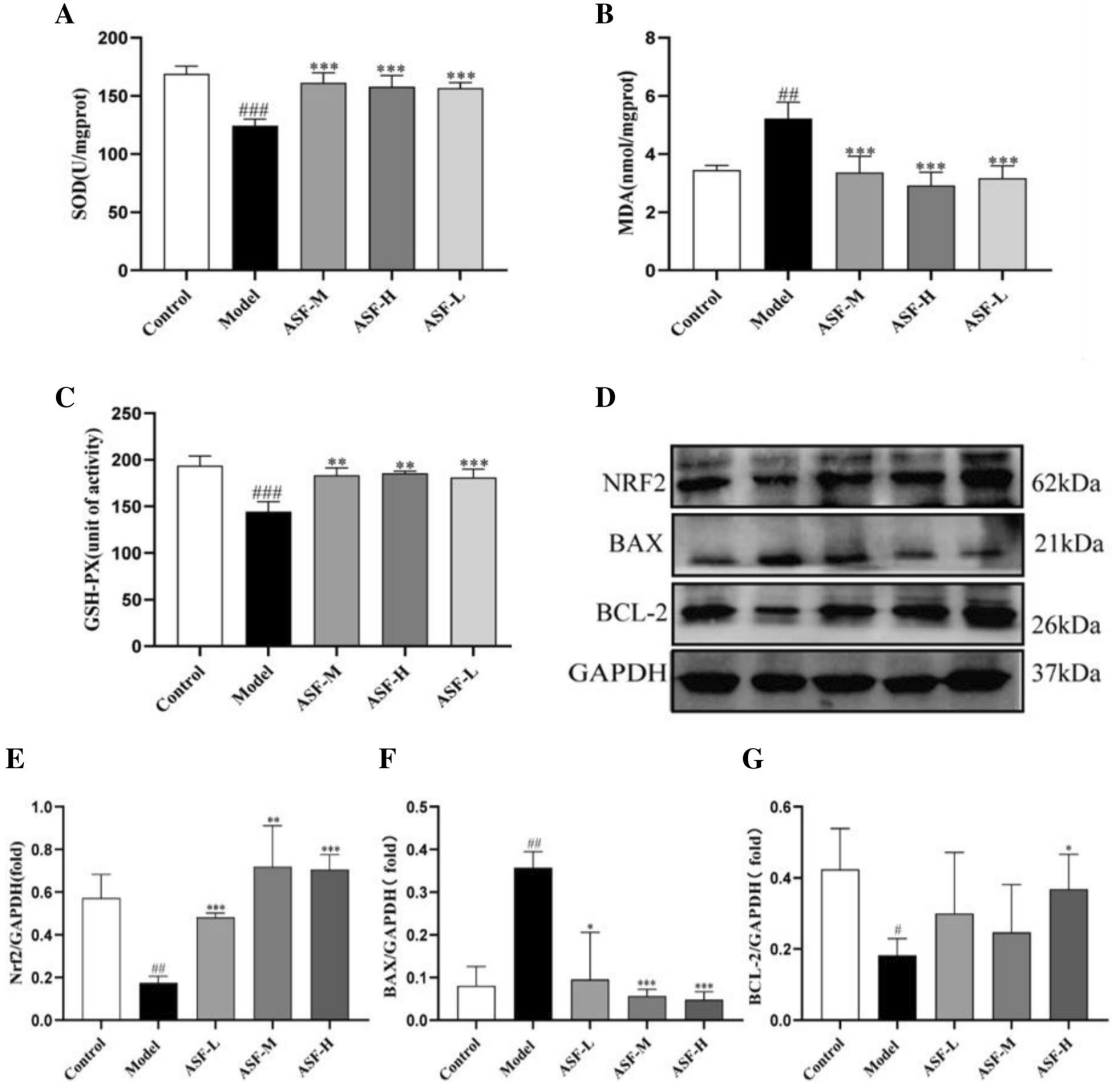
Neuroprotective effect of ASF extracts on oxidative stress and apoptosis in scopolamine-treated mice. The activities of SOD (**A**), the level of MDA (**B**) and the activities of GSH-Px (**C**) were determined by commercial assay kits; Representative western blotting images (D) and fold changes in relative densitometric values (**E**, **F**, **G**) of Nrf2, Bax, Bcl-2 were determined by western blotting assays. (n = 3). Data were analysed as mean ± SE (n = 3). ^#^*p* < 0.05, ^##^*p* < 0.01 and ^###^*p* < 0.001 compared to the control group; *p < 0.05, **p < 0.01 and ***p < 0.001 compared to the scopolamine-treated group

Continuously, we evaluate the possible antioxidative and antiapoptotic effects of ASF in mice. Firstly, Superoxide dismutase (SOD), Glutathione peroxidation enzyme (GSH-PX) and Malondialdehyde (MDA) were assessed as the important indicators reflecting the imbalance of the oxidation system. It can be seen from the Fig. 5 that the activity level of SOD (Fig. 5A) and GSH-PX (Fig. 5B) were significantly reduced and the content of MDA (Fig. 5C) was increased in hippocampus tissue of the scopolamine-treated group. While these changes were remarkably reversed by the ASF extracts administration with three doses (37.5 mg/kg, 75 mg/kg and 150 mg/kg). Moreover, as dose increased, the functional efficacy of ASF extracts in inhibiting AD increased.

Nrf2 is a crucial transcription factor, which exhibit antioxidative capacity, are known to plays a pivotal role in AD pathology, Thus, the Nrf2 expression was detected by western blotting. Then, we found an expected change in the expression level of Nrf2 protein in response to ASF treatment (Fig. 5D, E). The changes in Nrf2 expression were consistent with the concentrations of MDA and SOD, respectively, indicating that ASF reversed scopolamine-induced oxidative stress damage in mouse brain tissue.

In addition, many evidence suggested that the imbalance of antioxidant enzymes is accompanied by a large amount of protein and lipid peroxidation, which eventually leads to cell apoptosis [38]. To test if ASF-mediated mitigation of oxidative stress further led to decreased of cellular apoptosis. We assessed the expression levels of apoptosis-related protein. Bcl-2, a key member of the anti-apoptotic Bcl-2 family, which can inhibit the release of Cyt c into the cytoplasm and the formation of lipid peroxide. Bax is a central mediator of apoptosis that works in the opposite way to Bcl-2, which can promote apoptosis by inducing depolarization [39, 40]. Furthermore, the pro-apoptosis protein Caspase-3, a key enzyme in the mammalian apoptotic pathway, is unique in its effect on apoptosis of neuronal cells, which has been reported to be expressed at high levels in the AD brain [41].

As the results shows in Fig. 5F, G, compared with control group, the protein expression levels of Bax and Bcl-2 in the scopolamine-treated group were significantly decreased and increased, respectively, which were demonstrated by RT-qPCR and western blot. Interestingly, ASF treatment with high doses resulted in a significant reversal in the bcl2 and Bax protein expression levels.

### ASF inhibit the apoptosis of hippocampal neurons in scopolamine mice

**Fig. 6:**
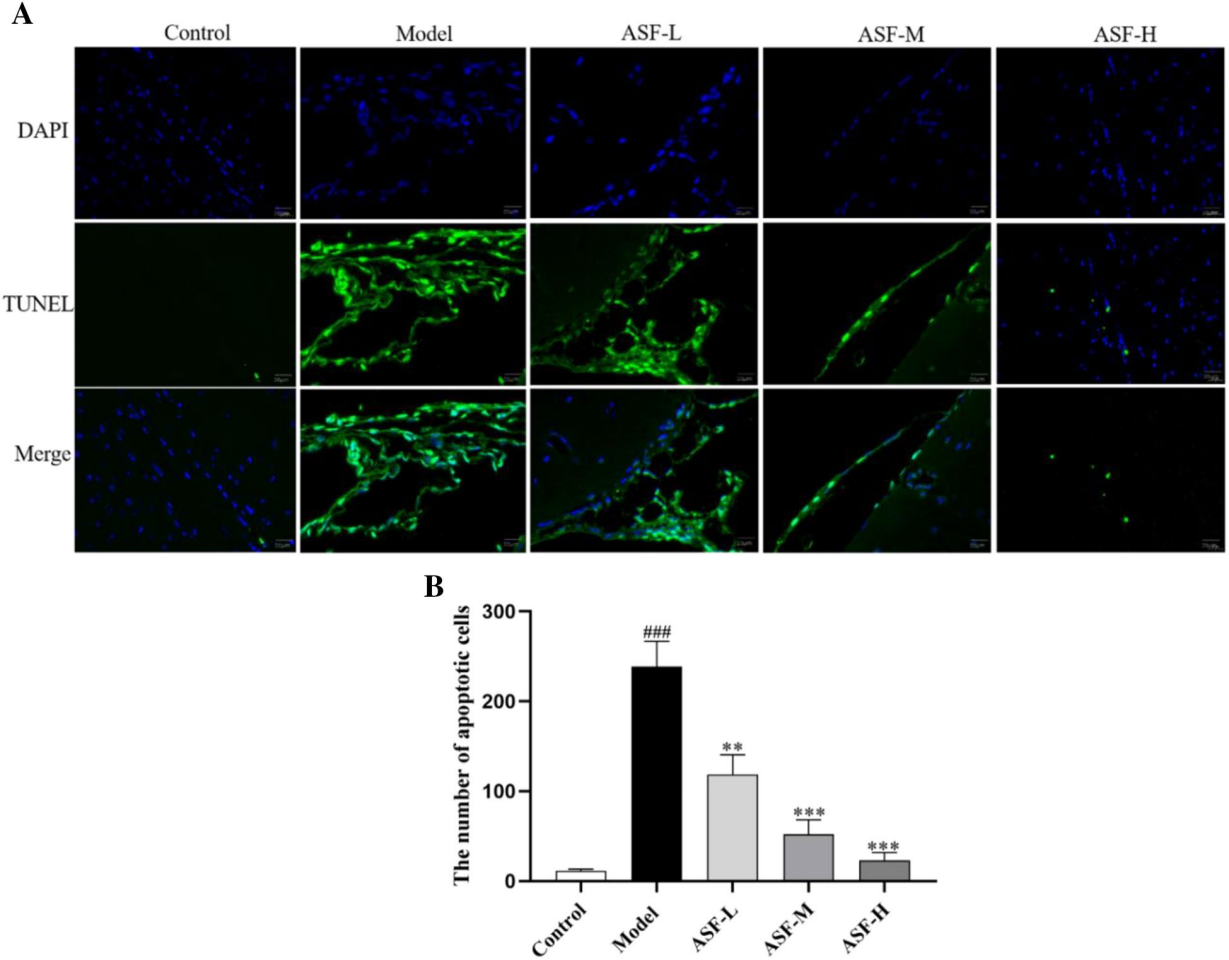
Neuroprotective effect of ASF extracts on apoptosis of hippocampal neurons. Representative images of TUNEL staining in each group (× 400) (**A**). Quantitative analysis of the apoptotic cells (**B**). Data were analysed as mean ± SE (n = 3). #*p* < 0.05, ##*p* < 0.01 and ###*p* < 0.001 compared to the control group; *p < 0.05, **p < 0.01 and ***p < 0.001 compared to the scopolamine-treated group

TUNEL staining was performed to further evaluate the effect of ASF on apoptosis of hippocampal neurons. Our results shown that there were significantly more apoptotic positive cells in the model group, showing the unique morphological characteristics of apoptotic cells. The number of TUNEL positive cells in the hippocampus of the ASF-treated mice was significantly reduced compared with the scopolamine model group (Fig. 6).

### ***ASF suppresses H***_***2***_***O***_***2***_***-induced oxidative stress in PC12 cells***

**Fig. 7:**
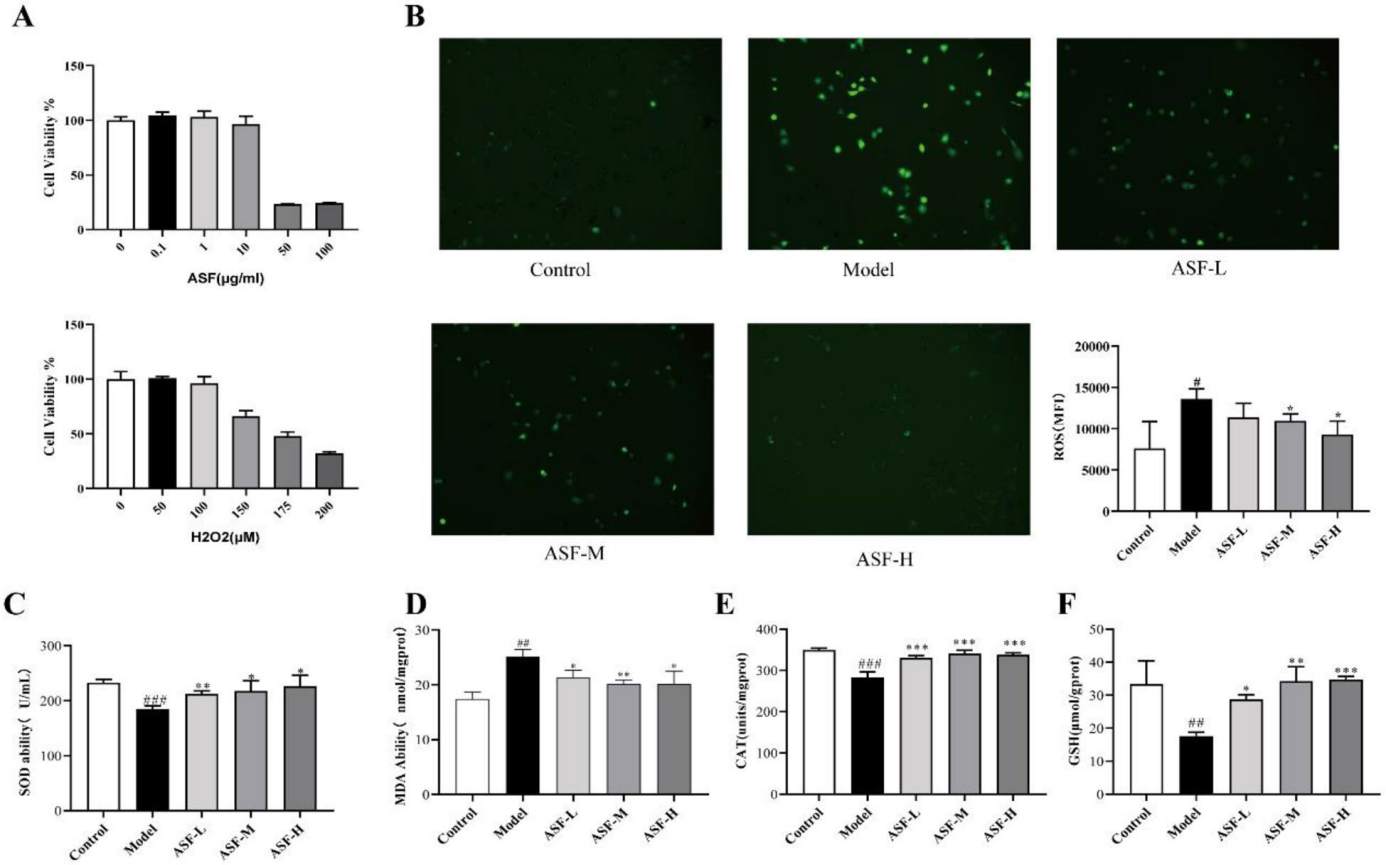
Effects of ASF on cell viability and H_2_O_2_-induced oxidative stress in PC12 cells. Cells were pre-treated with ASF (0.1, 1, 10ug/mL) for 24 h, and then stimulated with H_2_O_2_ (175uM) for 4 h. To assess the toxicity of ASF extracts and H_2_O_2_ to cells, we performed CCK8 assay in vitro (**A**). The ROS levels were detected by laser confocal microscopy (**B**). The levels of MDA (**D**) and activities of SOD, CAT, and GSH (**C**, **E**, **F**) in cells were determined by commercial assay kits. Data were expressed as mean ± SE (n = 3). ^#^*p* < 0.05, ^##^*p* < 0.01 and.^###^*p* < 0.001 significantly different from control group; *p < 0.05, **p < 0.01 and ***p < 0.001 significantly different from the H_2_O_2_- stimulated group

After we proved the neuroprotective effect of ASF extract in inhibiting oxidative damage and neuronal apoptosis in *vivo*, we processed a series of *vitro* experiments to investigate the potential mechanism of anti-AD mediated by ASF. We cultured PC12 cells and constructed an oxidative damage model stimulated by H_2_O_2_. To first determine the optimal intervention dose of ASF and the concentration of H_2_O_2_ for induction of cell damage, we assessed cell viability by CCK-8 assay. As shown in Fig. 7A, ASF showed no effect on the cell viability at 0.1-10ug/ml, Therefore, the ASF at concentrations of 0.1 μg/mL, 1 μg/mL, and 0.1 μg/mL as well as the 175 μM of H_2_O_2_ that are able to induce of about 50% cell death was chosen as the optimal working concentration for further experiments.

The imbalance of anti-oxidative stress system mediated by overload ROS is believed to associated with the AD process. Therefore, the scavenging effects of ASF on H_2_O_2_-induced oxidative stress was investigated. As shown in Fig. 7B, the fluorescence intensity of DCFH-DA displayed a significant increase in the PC12 cells stimulated with 175 μM of H_2_O_2_ for 4 h alone compared with the untreated control PC12 cells. Pretreatment of ASF with 0.1 μg/mL, 1 μg/mL and 10 μg/mL for 24 h before exposure to H_2_O_2_ resulted in a decreased ROS level. In addition, H_2_O_2_ stimulation caused a decrease in the levels of SOD, CAT and GSH (Fig. 7C, E, F), which was reversed by the three doses of ASF mentioned previously. As dose increased, the functional efficacy of ASF in inhibiting oxidative damage increased. while ASF suppressed the MDA level (Fig. 7D) induced by H_2_O_2_ in PC12 cells in a concentration-dependent manner. Hence, these findings contribute with important insight that ASF may be effective for AD treatment with an antioxidant dose-dependent effect.

### ***ASF resist H***_***2***_***O***_***2***_***-induced apoptosis of PC12 cells***

**Fig. 8:**
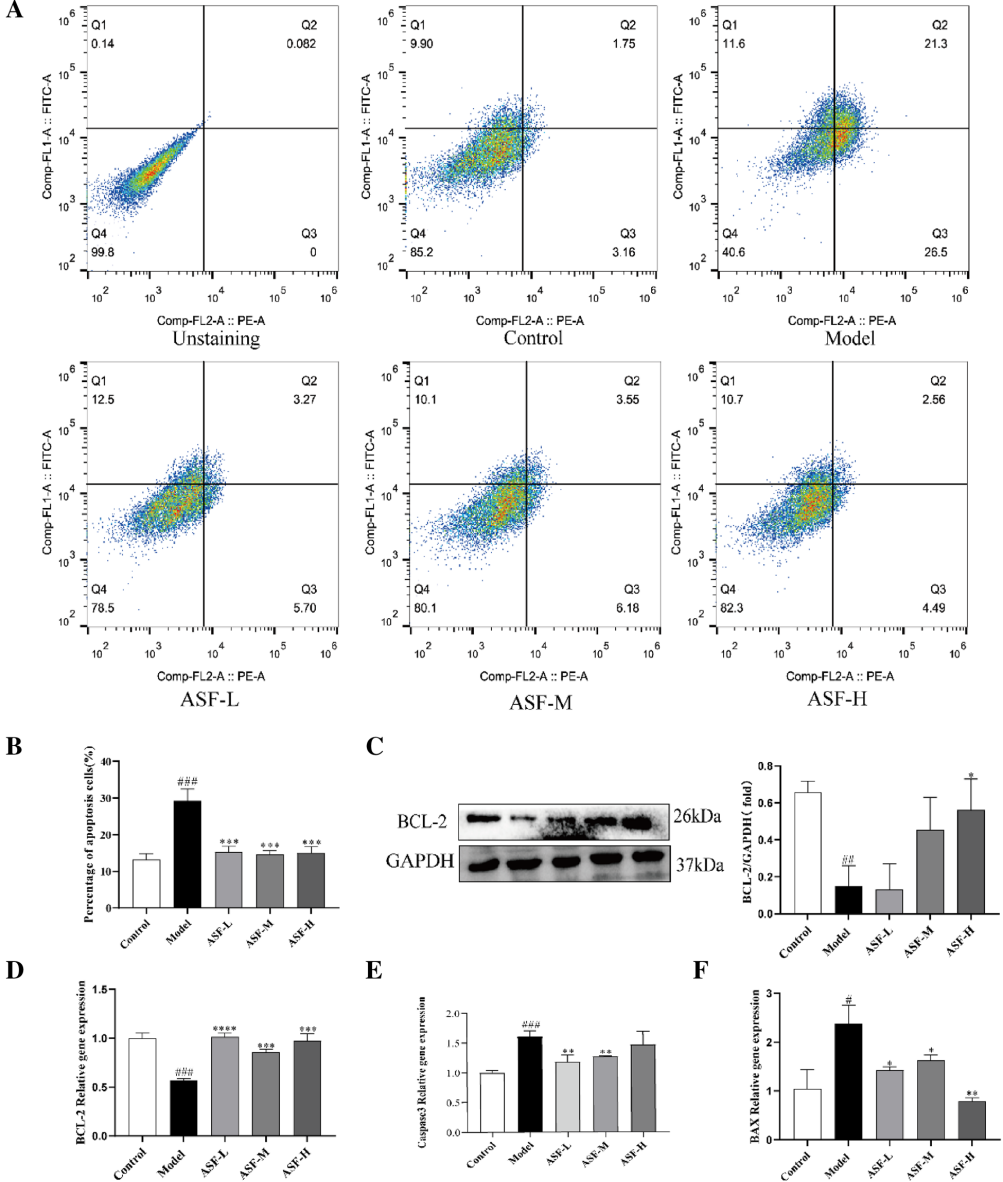
Effects of ASF on H_2_O_2_-induced apoptosis of PC12 cells. The percentage of apoptotic cells was measured by flow cytometry upon treatment with different concentrations of ASF for 24 h and subsequently exposure in H_2_O_2_ (175 μM)-treat for 4 h (**A**, **B**). Protein levels of Bcl-2 were analysed by Western blot (**C**). The gene expression levels of Bcl-2 (**D**), Caspase-3 (**E**), Bax (**F**) by RT-qPCR. Data were analysed as mean ± SE (n = 3). ^##^*p* < 0.01 and.^###^*p* < 0.001 versus control group; *p < 0.05, **p < 0.01 and ***p < 0.001 versus H_2_O_2_-stimulated group

Then, the rate of apoptosis and the expression of apoptosis-related proteins in PC12 cells subjected to H_2_O_2_ were detected. Based on the flowcytometry result, the apoptotic cell ratios of PC12 (11.6 ± 21.3%) were increased under the intervention of H_2_O_2_, compared to the control groups (9.9 ± 11.75%) (Fig. 8A, B). Whereas, ASF with different doses significantly decreased the percentage of apoptotic PC12 cells induced by H_2_O_2_. Furthermore, the evaluation of the anti-apoptotic effect of ASF on H_2_O_2_-induced cell apoptosis were assessed by qPCR and Western blotting. The results displayed a reduction in Bcl-2 protein expression and mRNA levels (Figs. 8C, 7D), and an increased Caspase-3 (Fig. 8E) and Bax mRNA levels (Fig. 8F) under H_2_O_2_ stimulation. Pretreatment with ASF (0.1, 1, 10 μg/mL) upregulated the protein expression of Bcl-2, and downregulated the expressions of Bax, caspase-3 compared to model PC12 cells. Our results further determine that the observed reduction in apoptosis may further resulted from the antioxidant effect of ASF.

### Identification of the constituents of ASF

**Fig. 9:**
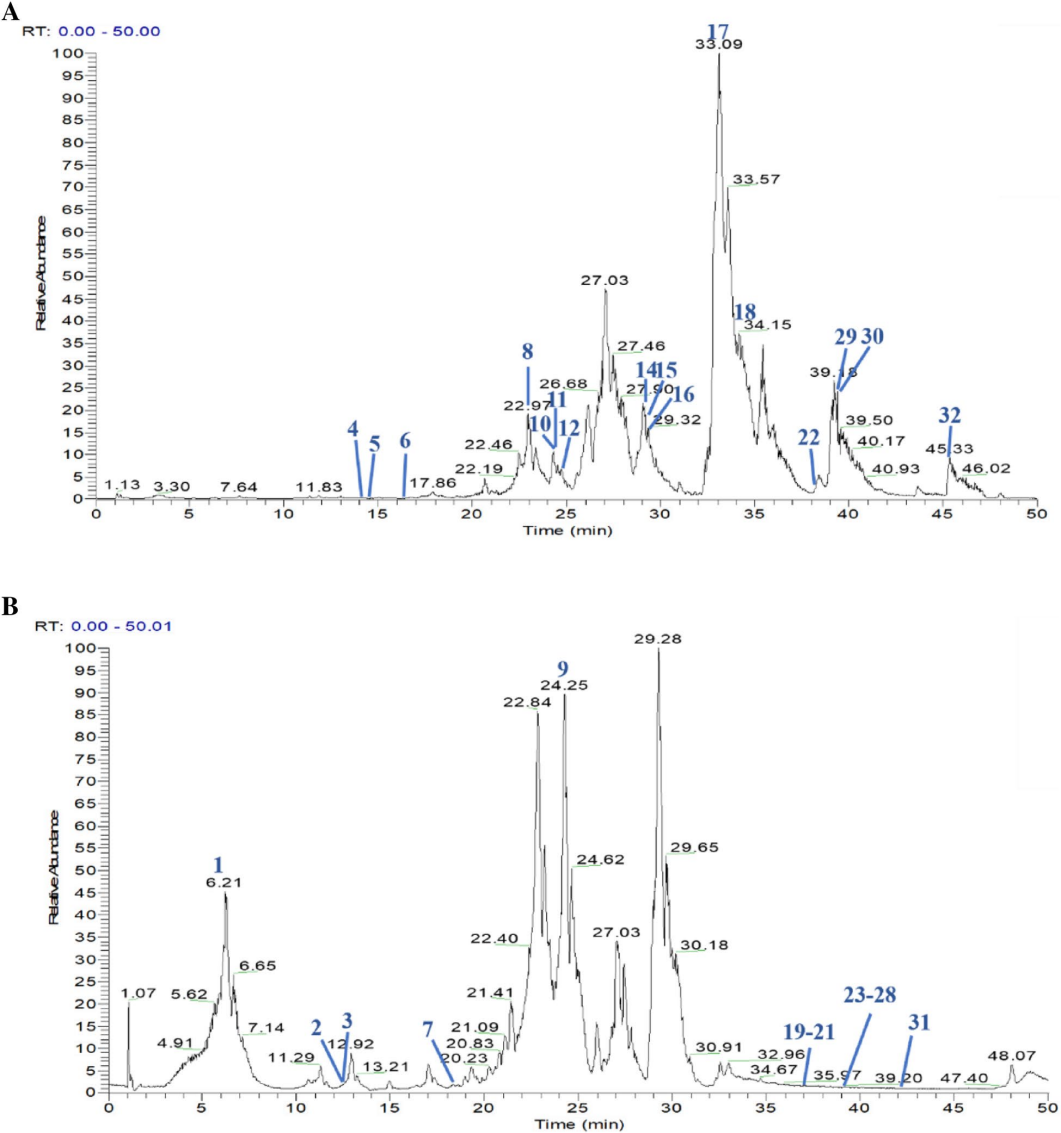
HPLC analysis identified constituents of ASF extracts. Total ion chromatogram monitored in positive (**A**) and negative (**B**) ion modes for ASF extracts

The chemical composition of the ASF extract was identified by UPLC-Q-Orbitrap MS and the ion current diagrams in positive and negative ion modes were obtained according to the chromatographic and mass spectrometric conditions. All the data were processed using Xcalibur version 2.7 software. We collected total 49 constituents of ASF based on the ETCM (http://www.tcmip.cn/ETCM/index.php/Home/Index/), TCMID (http://119.3.41.228:8000/tcmid/) and HERB (http://herb.ac.cn/). Then, comparing the molecular weight and structure of the published components, the major components were well separated and detected under optimized UHPLC and MS conditions (Fig. 9). Finally, 32 compounds were identified from ASF extracts including such as Farnesiferol C, Assafoetidnol B, Conferol, Farnesiferol A, Conferone (Additional file 2: Table S2).

### The integrated network pharmacology analysis indicated the potential targets mediated by Asafoetida in treating AD

**Fig. 10:**
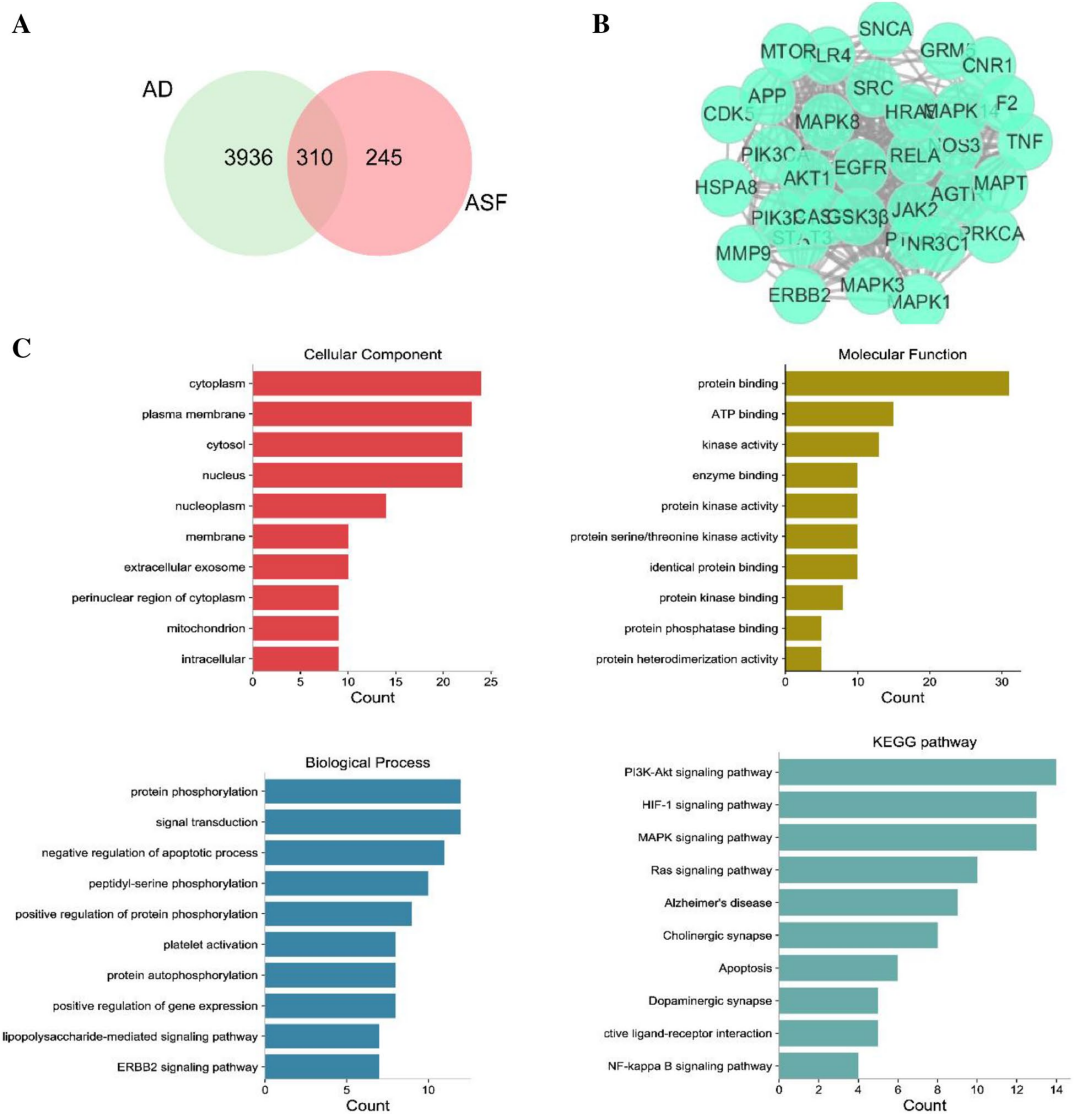
The integrated network pharmacology analysis indicated the potential targets mediated by ASF extracts in treating AD. The Venn diagram of the intersection of AD target and ASF target gene (**A**); Hub target genes of ASF extracts obtained from Protein–protein interaction (PPI) network based on three topological features (betweenness centrality, closeness centrality, and degree) (**B**); The cellular components (CC), molecular function (MF), biological process (BP), and KEGG enrichment analysis of targets mediated by ASF extracts (**C**), P < 0.05, the count of enrichment related targets decreases from top to bottom

Totally, 245 putative gene targets mediated by 32 identified constituents of ASF extracts were analyzed by Swiss Target Prediction. Meanwhile, a total of 4246 targets implicated in AD from GeneCards, OMIM database, DrugBank database, DisGeNET were eventually collected. After deleting duplicates, the intersection of putative ASF extracts targets and disease targets resulted in 310 hits by drawing Venn diagram (Fig. 10A). Then, the potential target genes of ASF extracts collected above were imported to the STRING database and the visualization of PPI network involved in core targets predicting was performed by cytoscape software 3.8.2. Through the screening of the set conditions, 33 hub targets were identified with potential in treating AD (Betweenness Centrality > 0.005020266, Closeness Centrality > 0.501644737, Degree > 38) (Fig. 10B). Then, the 33 targets were mapped to 79 items of cellular component (CC) mainly involved in cytoplasm, plasma membrane, cytosol, 218 items of molecular function (MF) mainly relate to protein binding, ATP binding, kinase, 2254 items of biological process (BP) closely connected with Protein phosphorylation, signal transduction, negative regulation of apoptotic process. And the top10 terms with high gene counts were shown in Fig. 10C. Using the KEGG enrichment analysis database, 73 pathways were retrieved, where unrelated pathways, such as "pathways in cancer", were excluded. We showed the top 10 pathways that met the criteria (p-value < 0.05, count > 8), of which PI3K/AKT signaling pathway is the best discriminant pathway of target enrichment.

**Fig. 11:**
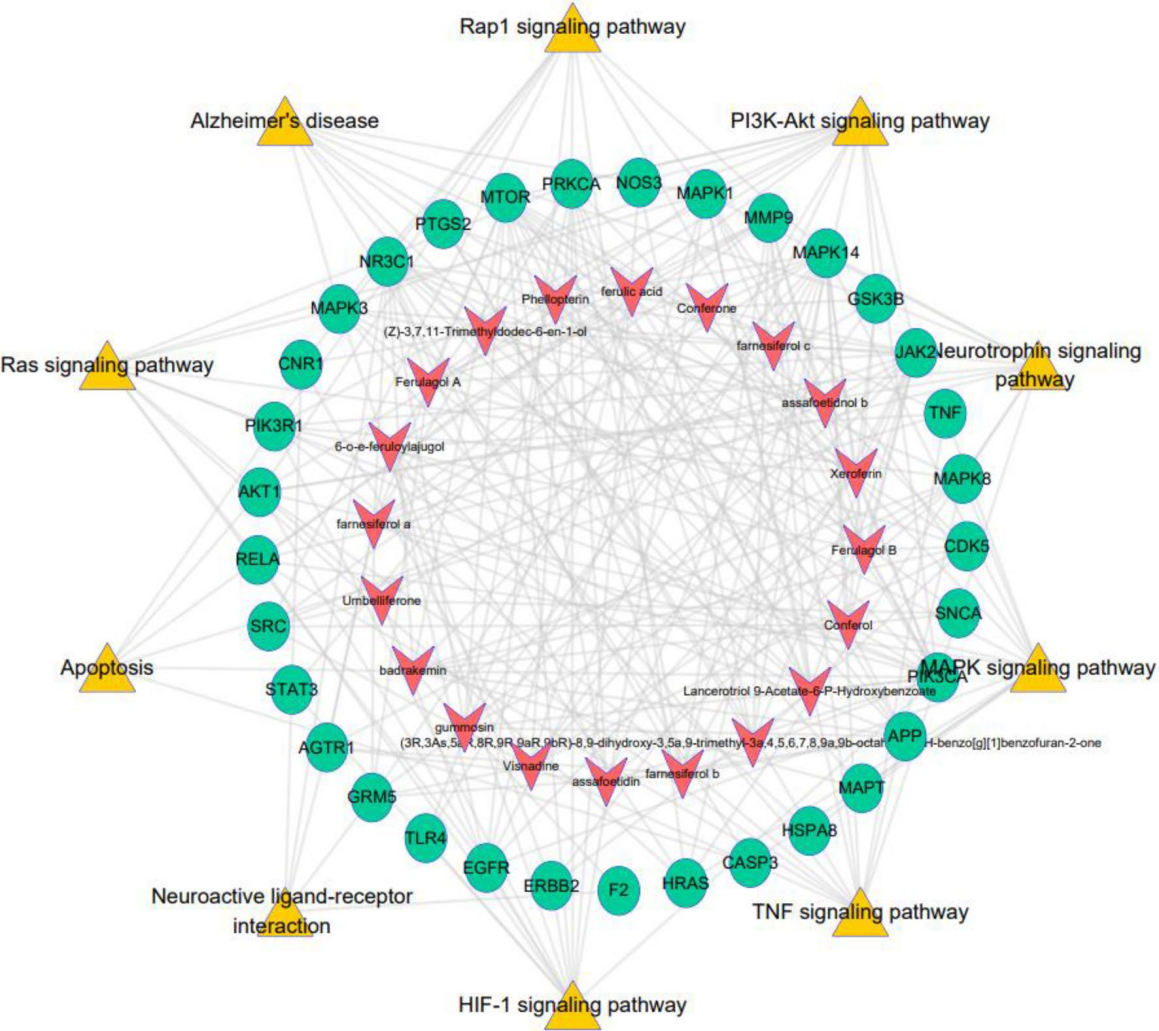
ASF extracts–constituent–AD related targets–pathway network. Orange triangle shapes represent the first 9 pathway in treating AD. The green circle shapes indicate 33 pivotal targets of ASF for AD treatment. Red arrow shapes represent the main constitutes of ASF corresponding to the 33 target genes

Next, to further clarify the action mechanism that main involved in the anti-AD effects of ASF extracts, the “constituents- common targets-pathways” network was constructed based on the above constitutes, targets, and signaling pathways information. As seen from Fig. 11, the top 20 active constituents corresponding to 33 target genes closely associated with disease progression were considered as critical components of ASF. PI3K/Akt pathways with the highest number of connections to target nodes were considered to be the main biological pathways.

### ASF protects against oxidative stress induced apoptosis through regulating PI3k/Akt/GSK3β/NRF2/HO-1 Pathway

**Fig. 12:**
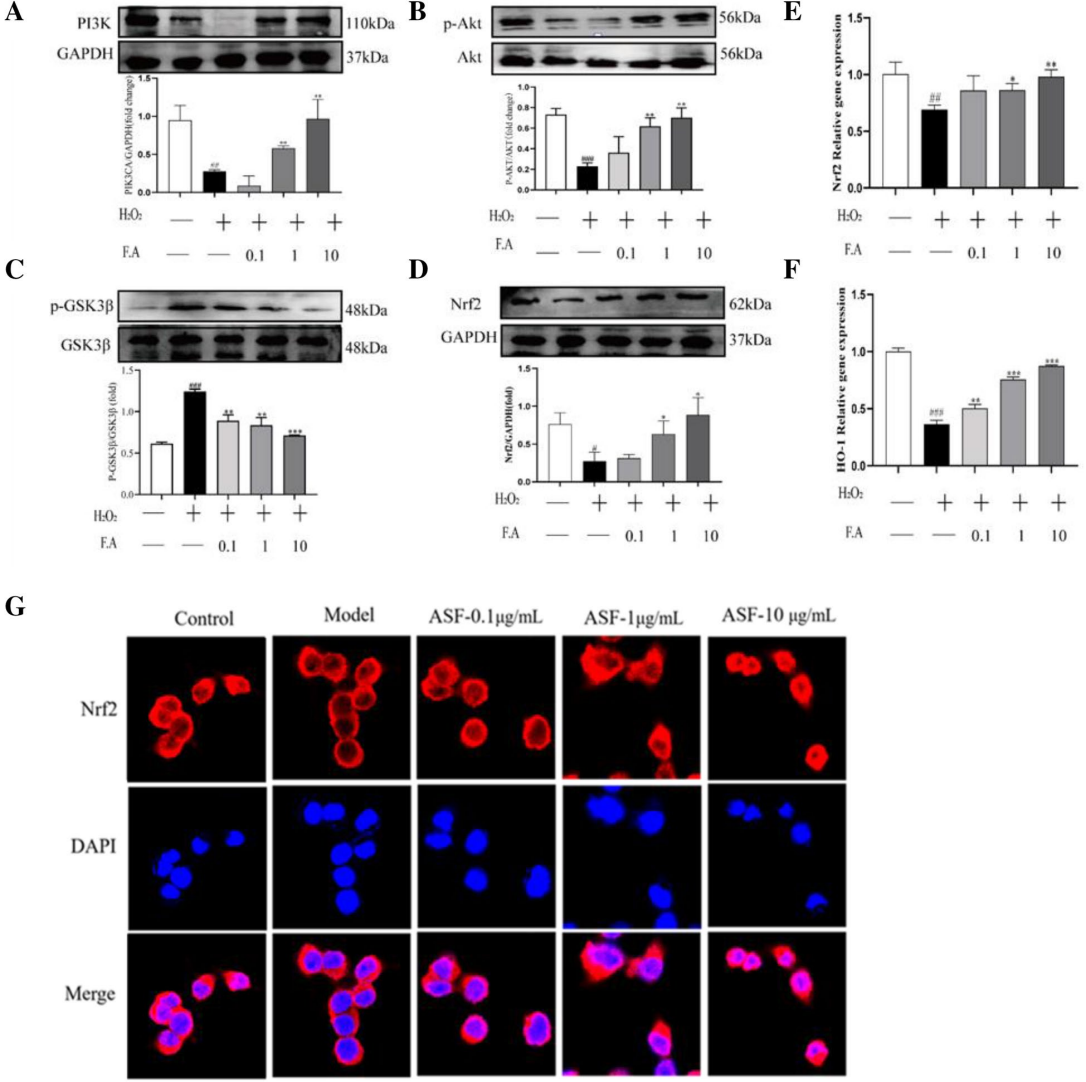
ASF promote the activation of PI3K/Akt/GSK3β/Nrf2/HO-1 signaling pathway in PC12 cells. Cells were pre-treated with ASF (0.1, 1, 10ug/mL) for 24 h, continuously were exposure to H_2_O_2_ (175uM) for 4 h. The expression PI3K, phosphorated Akt, Akt, phosphorated GSK3ß, GSK3ß, Nrf2 (**A**, **B**, **C**, **D**) were determined by western blotting assays (n = 3). Relative mRNA expression of Nrf2 (**E**) and HO-1 (**F**) in H_2_O_2_-treated PC12 cells were detected by real time-PCR analysis (n = 3). The nuclear translocation of Nrf2 was observed by laser confocal microscopy (**G**). Data were expressed as mean ± SE (n = 3). #*p* < 0.05, ##*p* < 0.01, ###*p* < 0.001 vs control group; *p < 0.05, **p < 0.01 and ***p < 0.001 vs H_2_O_2_-treated group

The potential targets and pathway of ASF extracts in mediating AD were further verified by western blot, qPCR and immunofluorescence staining in *vitro*. The PI3K/Akt is a typical anti-apoptotic signaling pathway. The publications suggest that its activation participates in the regulation of redox reactions and plays a pivotal role in protecting cells against oxidative stress [42] Glycogen synthase kinase 3 beta (GSK3β) is a serine/threonine kinase enzyme that controls neuronal functions. Its phosphorylation further involved in the regulation of different neuronal processes during AD pathogenesis such as cell death, axonal transport, cholinergic function [43]. Additionally, GSK3β can significantly down-regulate the expression of nuclear factor (erythroid-derived 2)-like 2 (Nrf2) and its downstream key antioxidant molecules Heme oxygenase-1 (HO-1). Therefore, we evaluated the modulatory effect of ASF on the PI3K/Akt /GSK3β/NRF2/HO-1 pathway in PC12 cells. The results from Western blot assay indicated that the decreased protein expression level of PI3K (Fig. 12A), phosphorylation AKT (P-AKT) (Fig. 12B), over-activated phosphorylation GSK3β (P-GSK3β) (Fig. 12C) and reduced Nrf2 protein expression level (Fig. 12D) in the PC12 cells stimulated by H_2_O_2_ were promoted by ASF treatment. Moreover, there were similar results in the mRNA expression level of Nrf2 and HO-1 in response to ASF treatment (Fig. 12E, F). Correspondingly, we found that Nrf2 was primarily located in the cytoplasm in normal PC12 cells. Compared with the H_2_O_2_-stimulated group, ASF facilitated a concentration-dependent increase of the nuclear translocation of Nrf2 following H_2_O_2_ exposure in PC12 cells, as proved by immunofluorescence staining (Fig. 12G). Collectively, these results indicated that the activation of the PI3k/Akt/GSK3β/NRF2/HO-1 signaling pathway contributes to the protective effect of ASF in preventing PC12 cells from oxidative stress induced apoptosis.

## Discussion

As more AD mechanisms are discovered, considerable research effort has been devoted to developing therapeutic strategies for treating AD. TCM is gaining attention as a potential treatment option for AD. Importantly, published literature and clinical trials provide evidence for the advantages of TCM in the treatment of chronic diseases due to their relatively fewer side effects compared with synthetic drugs [44]. To our knowledge, the potential therapeutic role of ASF in AD has not been investigated previously. In the present study, we firstly elaborated the pharmacological action of ASF in treating AD and further explored the underlying molecular mechanisms corresponding to the neuroprotective effects of ASF. Based on the scopolamine-induced cognitive impairment, we observed the behavioral phenotype of AD model mice and found that ASF effectively repair the cognitive impairment induced by scopolamine, as evidenced by improving memory and learning ability, reducing the pathological injury of hippocampal neurons, increasing the level of Ach, decreasing the activity of AchE. In addition, interestingly, in our research, we also found that ASF has the function of down-regulating oxidative stress and cell apoptosis in the brain of mice.

Oxidative stress, caused by the product of excess ROS, has been shown to be related to many diseases. These free radicals can exceed the scavenging ability of cell molecules, and this damage is particularly obvious in the brain due to high oxygen consumption [45]. In neurological diseases, elevated intracellular ROS levels could induce further pathological changes to brain neurons, which in turn leads to cognitive dysfunction [46]. In addition, AD patients are usually accompanied by the loss of neurons in the brain, especially in the hippocampus, a large part of which is caused by cellular apoptosis. There are many reasons for AD cell apoptosis, of which the oxidative stress is an important factor [47]. Studies have shown that in AD disease, oxidative stress can interfere with the progress of mitosis and disrupt the cell cycle, leading to cell apoptosis [48]. Therefore, we have further evaluated the effects of ASF on oxidative damage and neuronal apoptosis of the hippocampus in AD mice, and our results showed that ASF attenuates the scopolamine-induced decrease in Bcl-2 and the increase in Bax protein expression in mouse hippocampus, especially the high-dose group. This suggested that the ASF can perform anti-apoptotic effect in AD, and we speculate that one possible reason for this effect is the elimination of ROS by ASF. Afterwards, the determination of ROS scavenging enzyme and the final products of lipid peroxidation including SOD, GSH-PX and MDA verified our hypothesis. After understanding that the protective effects of ASF of reducing apoptosis and oxidative stress in *vivo*. We next processed a series of *vitro* experiments to clarify the biological mechanism responsible for the neuroprotective effects of ASF. Here we employed H_2_O_2_-treated PC12 cells, a commonly used cellular model for studying the underlying mechanisms of drug candidates with antioxidant potential. Consistent with the in *vivo* findings, we observed in *vitro* that the cellular ROS and MDA levels were significantly decreased and the activities of SOD, CAT, GSH in PC12 cells was increased after the exposure of H_2_O_2_, which was reversed by the administration of ASF. As dose increased, the functional efficacy of ASF in inhibiting oxidative damage increased. Furthermore, the elevated apoptotic rate in H_2_O_2_-treated PC12 cells as confirmed by flow cytometry analysis can be rescued by ASF. Similar improvements were also found in the expression of apoptosis-related proteins. Conversely, these results indicate that ASF exerts neuroprotective effects by improving oxidative stress and neuronal apoptosis, thereby slowing down the process of cognitive impairment.

Mechanically, we applied network pharmacology analysis and found that PI3K/Akt pathway is a highly enriched pathway. PI3K/Akt pathway is a multifunctional typical signaling pathway related to cell proliferation, anti-apoptosis and cell defense [49]. Studies have found that the activation of PI3K/Akt has a significant neuroprotective effect on neuronal damage. Glycogen synthase kinase (GSK)-3β is a key element downstream of the PI3K/Akt pathway and functions to regulate cell survival activity and the phosphorylation mediated by AKT can inhibit its expression. It has been documented that those compounds with GSK3β inhibitory activity seem to be an effective pharmacological approach for treating AD as they reduced neuropathological hallmarks and alleviated cognitive dysfunction in the in vivo model of AD [50]. In addition, the down-regulation of GSK-3β can also promote the separation of downstream Nrf2 and Keap1 [51]. Nrf2 pathway is a key way for cells to resist oxidation and maintain their own homeostasis [52]. And these free Nrf2 will continue to accumulate and then transfer to the nucleus, thereby further promotes downstream genes encoding antioxidant enzymes, and ultimately reduce oxidative damage. Therefore, we next verified this pathway. In vitro experiments, ASF can significantly up-regulate the expression of PI3K and further increase the phosphorylation of Akt, down-regulate the phosphorylation level of GSK-3β, and increase the protein expression level of Nrf2 and promote its entry into the nucleus to function. The increase in the expression level of HO-1 detected in our experiments (Additional file 3: Figure S1) also confirmed this point. Collectively, these results indicate that ASF can promote neuronal damage in the AD and slow down oxidative stress induced apoptosis through regulating PI3k/Akt/GSK3β/Nrf2/HO-1 Pathway. Moreover, our analysis of HPLC and comprehensive pharmacology identified the constituents of ASF extracts and the potential AD-related targets mediated by AD extracts. Additional molecular docking and molecular dynamics simulations provide insights into the binding stability of the main components of ASF and verified main protein targets. However, future in-depth mechanistic studies of ASF are necessary.

## Conclusion

Our work first uncovered the significant neuroprotective effect of ASF in treating AD in *vivo*. Then, the data presented in vitro prove that ASF can protect PC12 cells from H2O2-induced oxidative stress and secondary apoptosis. Furthermore, we elucidate that the neuroprotective effect of ASF may be through the regulation of PI3K/Akt/GSK3β/Nrf2/HO-1 pathway. Collecting together, our further evidence determined the great potential of ASF as a therapeutic agent against progressive AD. It presented a new promising for future AD treatment.

## Supplementary Information

**Additional file 1: Table.S1.** PCR primers of animal and cell.

**Additional file 2: Table.S2. **Analysis of the chemical constituents of ASF by UHPLC-Q-Orbitrap in positive and negative ion modes.

**Additional file 3.** The original images of Western blotting. **Figure S1.** The images for Western blot represent the expression level of PI3K,P-AKT,AKT,PGSK3β, GSK3β,Nrf2,Bcl2,Bax and the loading control GAPDH.

## Data Availability

The data generated in this study are available from the corresponding author upon request.

## Abbreviations

TCM: Traditional Chinese medicine
AD: Alzheimer’s disease
ASF: Asafoetida
MWM: Morris water maze
SDA: Step-down passive avoidance
NOR: Novel object recognition
SOD: Superoxide dismutase
MDA: Malondialdehyde
ROS: Reactive oxygen species
CAT: Choline acetyltransferase
GSH-PX: Glutathione Peroxidase
Ach: Acetylcholine
AchE: Acetylcholinesterase
GSH: Reduced glutathione
CCK8: Cell counting Kit-8
HO-1: Heme oxygenase-1
NOX: NADPH-oxidase
PPI: Protein–protein interaction
GSK-3β: Glycogen synthase kinase 3 beta
GO: Gene ontology
BP: Biological process
MF: Molecular function
CC: Cellular components
KEGG: Kyoto Encyclopedia of Genes and Genomes
RMSD: Root mean square deviation
RMSF: Root mean square fluctuation
