# Supplementary material for: Asafoetida exerts neuroprotective effect on oxidative stress induced apoptosis through PI3K/Akt/GSK3β/Nrf2/HO-1 pathway
Source: Chin Med. 2022 Jul 6;17:83. doi: 10.1186/s13020-022-00630-7 (PMC9258148; doi:10.1186/s13020-022-00630-7)
Supplement: Supplementary file 3 — Additional file 3. The original images of Western blotting. Figure S1. The images for Western blot represent the expression level of PI3K,P-AKT,AKT,PGSK3β, GSK3β,Nrf2,Bcl2,Bax and the loading control GAPDH. [file 13020_2022_630_MOESM3_ESM.pdf]

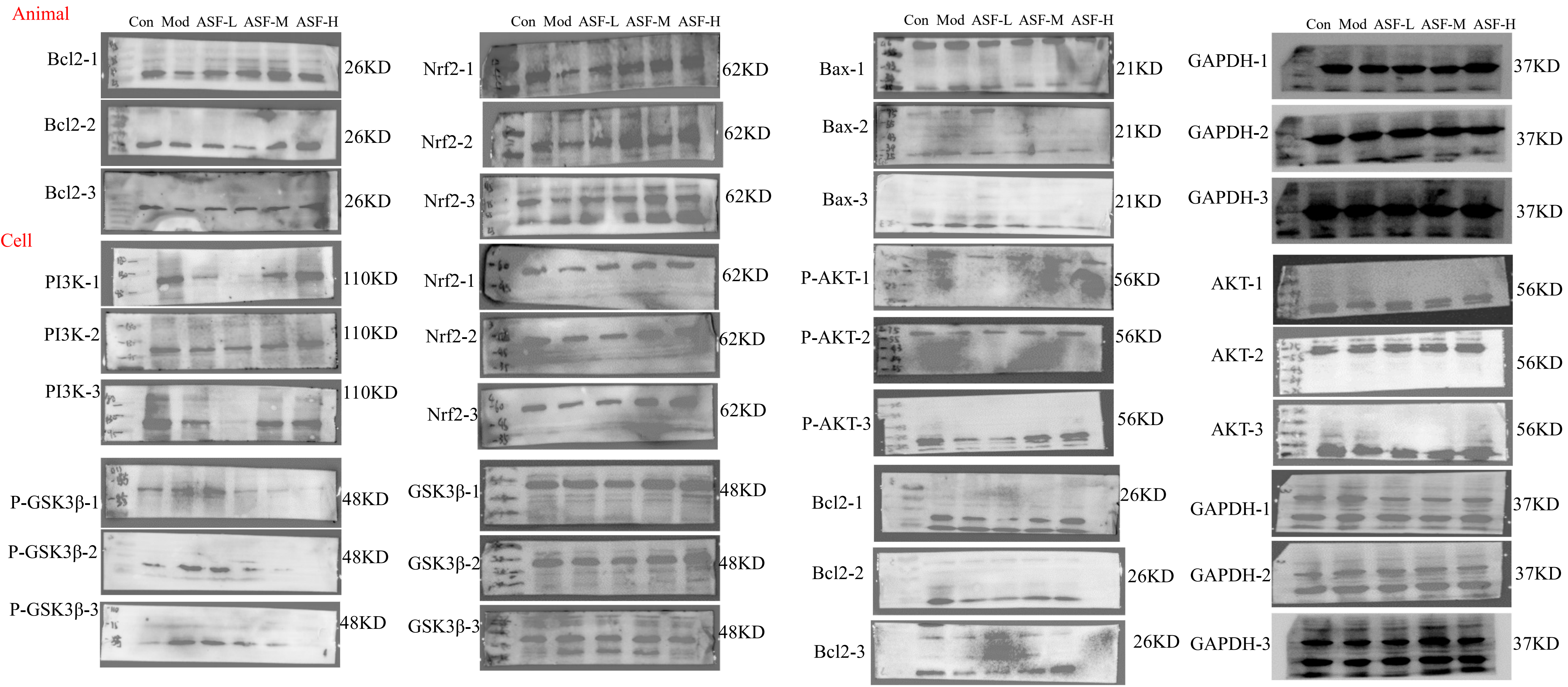

FigureS1:The images for Western blot represent the expression level of PI3K,P-AKT,AKT,P-GSK3β,GSK3β,Nrf2,Bcl2,Bax and the loading control GAPDH
